# Health-related quality of life in critically ill survivors: specific impact of cardiac arrest in non-shockable rhythm

**DOI:** 10.1186/s13613-021-00939-w

**Published:** 2021-10-24

**Authors:** Guillaume Geri, Nadia Aissaoui, Gwenhael Colin, Alain Cariou, Jean-Baptiste Lascarrou

**Affiliations:** 1 Paris-Saclay University, Versailles, France; 2 INSERM UMR1018, CESP, Villejuif, France; 3 AfterROSC network, Paris, France; 4 Medical Intensive Care Unit, Georges Pompidou European Hospital, Paris, France; 5 Paris University, Paris, France; 6 Medical Intensive Care unit, Les Oudairies Hospital, La Roche Sur Yon, France; 7 Medical Intensive Care Unit, Cochin hospital, Paris, France; 8 INSERM U970, Team 4 Cardiovascular Epidemiology and Sudden Death, Paris Cardiovascular Research Center (PARCC), Paris, France; 9 Medical Intensive Care Unit, Hotel Dieu Hospital, Nantes, France

**Keywords:** Healt-related quality of life, Cardiac arrest, Intensive care

## Abstract

**Background:**

Intensive care has a strong impact on health-related quality of life (HRQOL). The specific impact of cardiac arrest in non-shockable rhythm is poorly known.

**Patients and methods:**

We gathered patients included in two randomized controlled trials (AWARE and HYPERION). The HYPERION trial included ICU-treated non-shockable cardiac arrest patients. The AWARE study included ICU patients requiring mechanical ventilation. We compared the 3-months HRQOL of these patients to those of a large sample of the French general population. Physical and mental dimension were compared. Multivariable linear regression was used to pick up factors associated with HRQOL.

**Results:**

72 and 307 patients of the HYPERION and the AWARE studies were compared to 20,574 French controls. ICU patients evidenced lower scores in all the SF-36 dimensions compared to the controls. Similar scores were observed in both HYPERION and AWARe trials. The physical component score was lower in patients from the HYPERION trial compared to those from the AWARE trials and to controls (38.6 [29.6-47.8], 35.4 [27.5-46.4] vs. 53.0 [46.0-56.7], $\hbox {p}<0.001$). After adjustment for age and gender, HYPERION and AWARE trial status were associated wit lower physical component score.

**Conclusion:**

Health-related quality of life of unshockable cardiac arrest survivors evaluated at 3 months was similar to ICU survivors and significantly lower than in individuals from general population, especially in the physical dimensions.

**Supplementary Information:**

The online version contains supplementary material available at 10.1186/s13613-021-00939-w.

## Background

Patients surviving critical illness suffer major decrease of health-related quality of life (HRQOL) compared to age- and gender- matched general population immediately after discharge [1–3]. Although this may recover over time, such impairment may last for months after ICU discharge, especially on physical components of HRQOL.

Besides the mid- and long-term physical complications, psychologicaland mental impairment may be also observed regardless the reason for admission to the ICU. This is all the more true in patients with brain injury, related either to trauma or to anoxic encephalopathy. In the latter, HRQOL of cardiac arrest survivors is described as moderately impaired compared to population norms [4–6]. However, the vast majority of cardiac arrest survivors included in these observational studies were resuscitated from initial shockable rhythm [7, 8] ; this could constitute a bias as brain injury is different in terms of pathogenesis and impairment in these patients compared to those resuscitated from non-shockable cardiac arrest [9].

While the beneficial impact of targeted temperature management on mortality [10, 11] and on functional outcomes as HRQOL [12] is now well accepted in shockable cardiac arrest patients, data are lacking in non-shockable cardiac arrest patients. Recently, moderate therapeutic hypothermia at $33^{\circ }\hbox {C}$ has been shown to increase survival with favorable neurological outcome in these patients [13]. Whether patients with favorable outcome expected alterations of their quality of life as compare to others survivors of critical care is unknown.

In the present study, we aimed to describe the 3-months HRQOL of non-shockable cardiac arrest survivors and to compare these results to ICU survivors and general population.

## Patients and methods

### Source of data

#### HYPERION Study

Data from cardiac arrest survivors of cardiac arrest in non-shockable rhythm were obtained from the HYPERION trial follow-up [13]. HYPERION trial was an investigator-initiated, blinded-outcome-assessor, parallel, two-arm, multicentre, randomised clinical trial conducted in 25 intensive care units (ICUs) in France between January 2014 and January 2018. The objective of the HYPERION trial was to assess whether, compared to targeted normothermia (37 $^{\circ }\hbox {C}$), targeted temperature management (TTM) at 33 $^{\circ }\hbox {C}$ improved the neurological outcome of comatose patients successfully resuscitated after cardiac arrest in a non-shockable rhythm due to any cause. The primary outcome was the proportion of patients with a favorable day-90 neurological outcome, defined as a Cerebral Performance Category (CPC) score of 1 (good cerebral performance or minor disability) or 2 (moderate disability) [14]. The CPC was assessed during a semi-structured telephone interview by a single psychologist specifically trained for the study and blinded to the treatment group at day 90 after admission. During this interview, several HRQOL scores were performed by the psychologist [15].

#### AWARE Study

Data from survivors of critical illness were obtained from the AWARE trial follow-up [16]. AWARE trial was an investigator-initiated, parallel, two-arm, multicentre, randomised clinical trial conducted in 46 ICUs in France between 2012 and 2014. The objective was to determine whether a strategy aiming to prevent oversedation could reduce 90-day mortality in critically ill patients requiring mechanical ventilation compared to usual care. The primary outcome was 90-day mortality after ICU admission and secondary outcomes included HRQOL scores assessed by psychologist during telephone interview at day 90.

#### Reference group

As a reference group, we used the most recent decennial survey on population health and medical services involving 25,000 families conducted in France by the National Institute of Statistics and Economic Studies (INSEE) between October 2002 and October 2003. Its main objective was to evaluate subjects’ perspective and behaviors regarding their health. The survey involved an estimate of annual health resources consumption, use of physicians’ services, purchase of medicines prescribed and not prescribed, as well as an investigation of subjects’ behavior towards medical prevention, diet and exposure to risk, in particular professional risks.

### Outcomes

The primary outcome was the HRQOL assessed by the French translated version of the Medical Outcomes Study Short-Form 36 Health Survey (MOS SF-36, v2) questionnaire [17] delivered at 3 month, which has been previously used with cardiac arrest patients. Briefly the SF-36 provides an assessment of physical and mental HRQOL [18]. This 36-items questionnaire evaluated eight dimensions of functioning and well-being (role limitations because of physical problems (“role-physical”), bodily pain, physical functioning, general health perception, vitality, social functioning, role limitations because of emotional problems (“role-emotional”) and mental health (psychological distress and psychological well-being)). Dimensions can be investigated separately but can also be combined to provide summary scores of physical and mental health, together with a total score (8 dimensions combined) [19]. Scores range from 0 to 100, with higher scores indicating better health. Physical and mental component summary scales (PCS and MCS) are then computed as weighted composites of the 8 scales (physical functioning, general health, bodily pain and role physical in PCS and social functioning, role emotional, mental health and vitality in MCS).

### Statistics

Descriptive statistics were reported as mean (with standard deviation) or median (with interquartile) and proportion (percentage) for continuous and categorical variables, respectively, unless otherwise specified.

Categorical variables were compared between groups using Pearson’s $\chi ^{2}$ test while continuous variables were compared using Mann-Whitney test. The comparison of the three groups altogether was performed using a Kruskal-Wallis test.

Association between physical component score and study was analyzed in a multivariable model adjusted on age and gender. Same analysis was rerun on mental component score. Missing values accounted for less than 6% of each dimensions of the SF36 questionnaire in all groups (Additional file 1: Figure S1).

All tests were two-sided with $\hbox {p}<0.05$ considered statistically significant.

We performed the analyses using R (R Core Team (2017). R: A language and environment for statistical computing. R Foundation for Statistical Computing, Vienna, Austria. URL https://www.R-project.org/.)

## Results

**Fig. 1:**
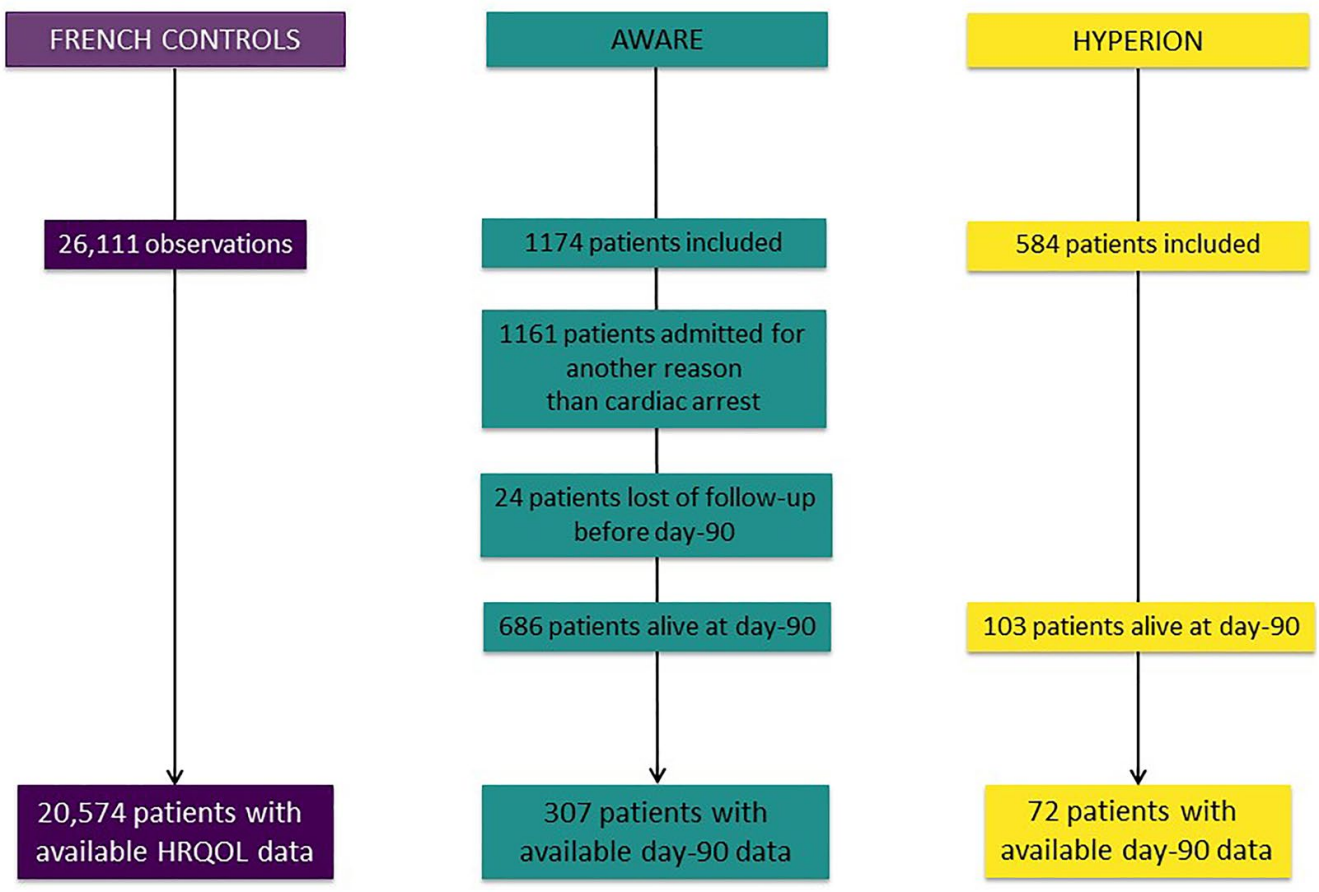
Flowchart of the study

Among the 584 and 1,174 patients included respectively in the HYPERION and the AWARE trials, 103 (17.6%) and 683 (58.2%) were alive at day 90. Among those, HRQOL data were available in 72 (70%) and 307 (45%) of them, respectively (Fig. 1). Of the 26,111 observations recorded in the INSEE study, HRQOL data were available in 20,574 (82.3%). Demographics of patients included, lost of –follow-up and dead before day-90 evaluation did not differ in the HYPERION trial while there were significant differences in demographics of these different subgroups in the AWARE trial (Additional file 1: Tables S1–S3).

**Table 1:** Baseline characteristics according to patients’ groups. Data are provided as median [interquartile] and n(%) for continuous and categorical variables, respectively

	AWARE	HYPERION	INSEE	p-value
	$\hbox {N}=307$	$\hbox {N}=72$	$\hbox {N}=20574$	
Age	62.0 [50.0;70.8]	64.0 [56.0;71.5]	44.0 [32.0;56.0]	$<0.001$
Male gender	184 (59.9%)	49 (68.1%)	9675 (47.0%)	$<0.001$
SAPS2 score	45.0 [35.0;56.0]	73.0 [55.0;80.0]	–	$<0.001$

Patients from HYPERION and AWARE trials were older as compared to control subjects (64 [56,72] and 62 [50,71] vs. 44 [32,56],* p* < 0.001)(Table 1). The vast majority of cardiac arrest patients included in the HYPERION trial collapsed in presence of witness and received bystander cardiopulmonary resuscitation. Most patients included in the AWARE trial were admitted for acute respiratory distress and septic shock (Additional file 1: Table S2). At the time of interview, the Cerebral Performance Categories (CPC) score of HYPERION patients was 1,2 and 3 in 25 (34.7%), 16 (22.2%) and 31(43.1%) patients, respectively. No difference in terms of CPC score was observed between survivors at day-90 included and not included in the present analysis. The CPC score was collected from the next-of-kin when the patient was unable to answer it.

**Fig. 2:**
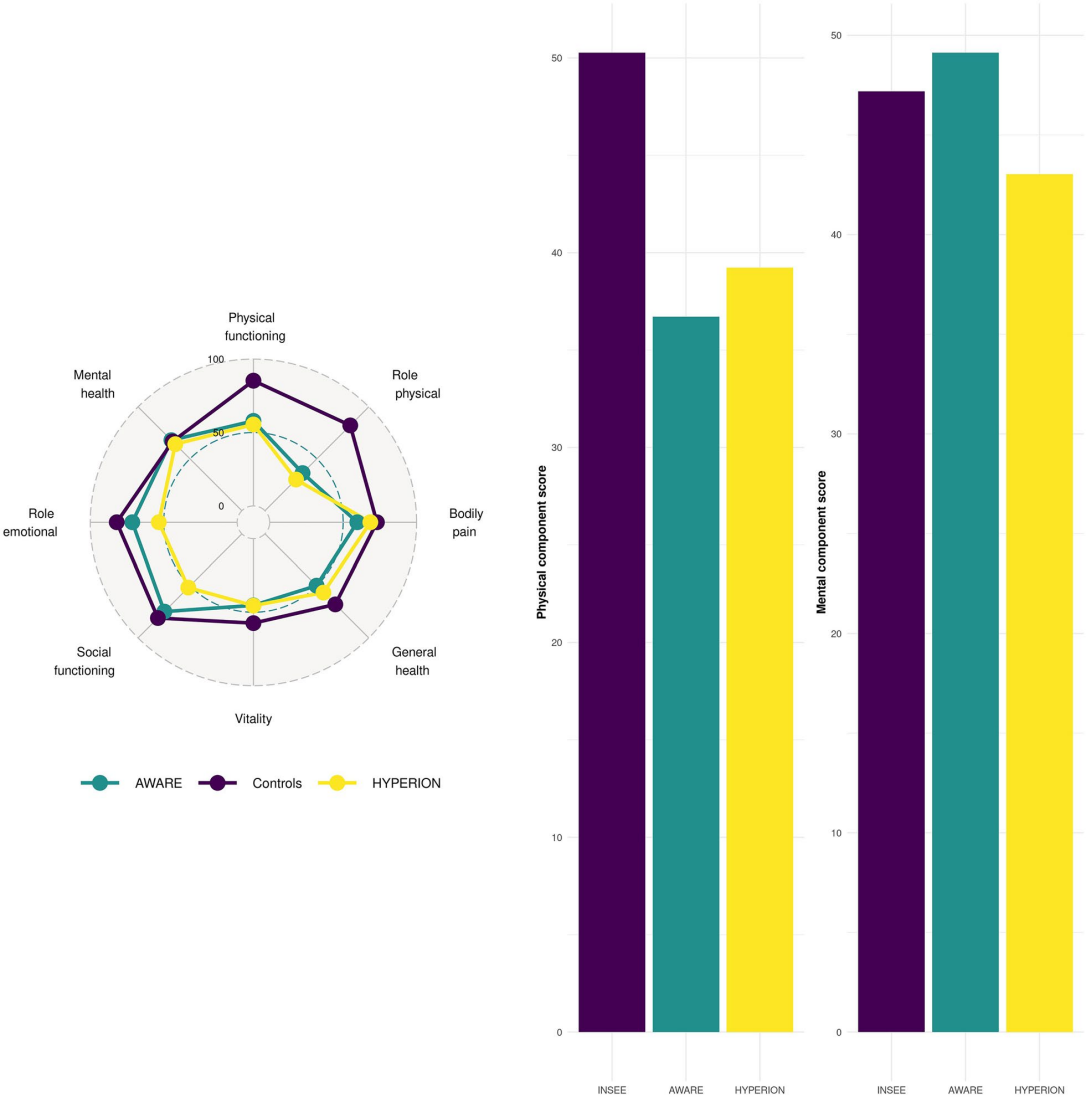
Health related quality of life of patients included in the AWARE and HYPERION trials and controls from the general French population. Panel A shows the mean scores of the three groups in the 8 dimensions of the SF-36 questionnaire. Panel B shows the mean physical and mental component scores of the three groups

**Table 2:** Results of HRQOL interview at day-90. Data are shown as median [interquartile] and compared using a Kruskal-Wallis test

	AWARE	HYPERION	INSEE	
	$n=307$	$n =72$	$n=20{,}574$	
Physical functioning	65.0 [35.0,85.0]	60.0 [20.0,85.0]	95.0 [80.0,100]	$<0.001$
Role physical	25.0 [0.00,75.0]	0.00 [0.00,75.0]	100 [75.0,100]	$<0.001$
Bodily pain	61.0 [31.0,100]	72.0 [41.0,100]	74.0 [52.0,100]	$<0.001$
General health	52.0 [32.0,67.8]	57.0 [45.0,72.0]	71.2 [57.0,82.0]	$<0.001$
Vitality	45.0 [30.0,60.0]	45.0 [25.0,62.5]	60.0 [45.0,70.0]	$<0.001$
Social functioning	87.5 [56.2,100]	50.0 [25.0,75.0]	87.5 [62.5,100]	$<0.001$
Role emotional	100 [33.3,100]	66.7 [0.00,100]	100 [66.7,100]	$<0.001$
Mental health	68.0 [56.0,80.0]	64.0 [52.0,78.0]	68.0 [56.0,80.0]	0.337
Component summary scales				
Physical (PCS)	35.4 [27.5,46.4]	38.6 [29.6,47.8]	53.0 [46.0,56.7]	$<0.001$
Mental (MCS)	51.8 [40.3,57.3]	44.6 [32.4,52.8]	49.5 [42.2,54.0]	$<0.001$

Patients included in the HYPERION trial evidenced similar scores in all the SF-36 dimensions compared to those included in the AWARE trial but both scored lower in the physical health dimensions (Fig. 2A; Table 2) compared to the controls. The physical component score was lower in patients from the HYPERION trial compared to those from the AWARE trials and to controls (38.6 [29.6,47.8], 35.4 [27.5,46.4] vs. 53.0 [46.0,56.7], $p<0.001$). The mental component score was lower in patients from the HYPERION trial but higher in those included in the AWARE trial compared to controls subjects (44.6 [32.4,52.8] and 51.8 [40.3,57.3] vs 49.5 [42.2,54.0];* p* value $< 0.001$, respectively) (Fig. 2B). The physical component score was significantly lower in HYPERION patients in the control arm (TTM37) compared to the intervention arm (TTM33) while no difference was observed regards to the mental component score (Additional file 1: Tables S4 and S5). After adjustment for age and gender, HYPERION and AWARE trial status were associated with lower physical component score but evidenced discordant associations regards to the mental component score as patients included in the AWARE study had increased MCS while patients included in the HYPERION study had decreased MCS compared to controls (Table 3).

**Table 3:** Factors associated with physical and mental component scores. Multivariable linear regression

	Coefficient( 95% conf. interv.)	*p*-value
Mental component score		
Age, per 10y-increase	0.10 [0.01, 0.17]	0.02
Male gender	2.64 [2.38, 2.90]	$<0.001$
Group of patients		
French controls	Ref	
AWARE study	1.50 [0.39,2.60]	0.008
HYPERION study	$-$4.89 [$-$7.16, $-$2.60]	$<0.001$
Physical component score		
Age, per 10y-increase	− 2.3 [− 2.4, − 2.3]	$<0.001$
Male gender	1.07 [0.85, 1.30]	$<0.001$
Group of patients		
French controls	Ref	
AWARE study	− 10.29 [− 11.23,− 9.35]	$<0.001$
HYPERION study	− 7.11 [− 9.05, − 5.17]	$<0.001$

## Discussion

In the present study, we compared 3-months health related quality of life of non-shockable related out-of-hospital cardiac arrest survivors to patients discharged alive from the intensive care unit and to general population. We observed that the physical component score was strongly impacted in critical care survivors while the mental component score was interestingly better in this subgroup compared to general population.

HRQOL was significantly decreased in patients discharged alive from the ICU, whatever the reason of admission, especially on the physical dimensions. Eggman et al reported very similar results in ICU survivors who experienced a significant decrease of the physical component score but almost no alteration of mental dimensions [20]. These results confirmed previously reported ones that evidenced altered HRQOL physical dimensions in ICU survivors admitted to the hospital for various reasons [21–24]. Accordingly and at a longer term, Herridge et al observed a clear impact of acute respiratory distress syndrome on physical dimensions of HRQOL of ICU survivors while this impact was minimal on mental dimensions compared to general population norms [25]. Interestingly, this negative impact improved over time to reach population norms after several years of follow-up. This could at least partly explain the apparent discordant results that have highlighted the lack of difference in terms of health related quality of life in ICU survivors [26, 27]. These discrepancies may also rely on the wide variety and severity of diseases that may be seen in the intensive care setting. Ehooman et al recently observed a marked decrease of HRQOL of critically ill survivors with hematological malignancies. Interestingly they compared patients with and without hematological malignancy and admitted to the ICU for septic shock: HRQOL was more deeply impaired in patients with than without hematological malignancy [28]. Such a finding suggest the weight of patients’ characteristics and pre-existing HRQOL to subsequent quality of life. Thus, the initial severity may be a strong predictor of mid- and long-term HRQOL. The SOFA score was associated with 6-months HRQOL in the results provided by Ehooman et al, as prolonged mechanical ventilation or extracorporeal life support had previously been associated with such a qualitative outcome in ICU survivors [29–31].

Accordingly, cardiac arrest is probably the most demonstrative disease in terms of severity, life-support therapies as well as neurological consequences of brain anoxic injury. In the present study, we observed a significant decrease of HRQOL physical component score compared to population norms. We already reported the monocentric follow-up of a cohort of out-of-hospital cardiac arrest survivors and observed an impact in terms of physical functioning compared to age- and gender-matched healthy individuals from the general population in a neurological impairment manner [4]. In other words, we did not observe any difference in terms of HRQOL in OHCA survivors who fully recovered (defined as Cerebral Performance Categories [CPC] at 1) while there was a significant alteration of HRQOL dimensions over increasing CPC categories. Such a finding - similar to previously reported ones - could be explain by the natural selection bias of the patients included in these observational studies. This is reinforced by the fact that there was in the present study a selection of patients with good neurological outcome among those who were alive at day-90. Indeed, while the CPC score was 27, 19, 53 and 1% (for categories 1, 2, 3 and 4, respectively) in patients alive at day-90, patients included in the present analysis rated CPC 1, 2 and 3 in 35, 22 and 43%, respectively. This reinforces the signification of our results as if we could have rated HRQOL in all patients alive at day-90, we could assume the impairment we observe in terms of physical dimensions ofHRQOL would have been larger. Most of the data published so far are indeed from patients resuscitated from shockable rhythm related cardiac arrest [5, 7]. Furthermore, this highlights the originality of the current data on patients reputed to be the sickest ones in the field of cardiac arrest.

Interestingly, while critically ill patients included in the AWARE study and discharged alive from the ICU evidenced better mental component scores than individuals from general population, we did not observe similar findings in the population of cardiac arrest survivors who had been included in the HYPERION trial. This difference remained significant after adjustment for age and gender. Such a finding may raise several hypothesis. As it has been previously suggested, ICU survivors may experience active coping strategies during recovery [32]. Indeed, ICU survivors may evidence some exaggerated feelings of joy over having survived or amnesia of the ICU stay. This is all the more plausible that most of the studies about the long-term follow-up of critically ill survivors reported similar results. Another contributing hypothesis could be that our findings illustrate the concept of “response shift”, also called the ‘disability paradox’ [33–35]. Response shift refers to a change in the meaning of one’s self-evaluation of HRQOL and could explain such a paradoxical finding. This has been well studied in oncology research [36, 37] as well as in spinal cord injury or stroke patients [38]. Taken together, this could reflect the fact that critically ill but cardiac arrest survivors are adapting to their condition. We could hypothesize that neurological impairment, event subclinical, in cardiac arrest survivors may act as a worsening factor in mental dimensions of HRQOL. Whatsoever, this encourages future research to take into consideration additional factors that could contribute to HRQOL such as return to work [39, 40] of functional disabilities in daily-life activities [4].

We acknowledge several limitations. First, due to the inclusion criteria of the HYPERION trial, we were not able to evaluate HRQOL in a representative cohort of OHCA survivors but only in unshockable rhythm related cardiac arrest. This is however the first report of HRQOL in this very specific subgroup of patients. Second, we provide an early evaluation of HRQOL as most of the reports published so far showed results of 6 or 12 months. Third, we did not assess HRQOL in a longitudinal way as it has been well demonstrated that such a patient-centered outcome could improve overtime. Fourth, HRQOL data were only available in 45% and 70% of AWARE and HYPERION patients alive at day-90. While characteristics of patients from the HYPERION study were similar between those included and not included in the present analysis, this was not the case for patients from the AWARE study. Thus, we cannot exclude our findings would have been different if we had collected data from lost-of-follow-up patients. Fifth, we were not able to provide indirect data impacting HRQOL such as return to work or re-hospitalization at day-90 because such data were not collected in both trials. Last, results have been obtained from exclusively French data, which might, despite their multicentric prospective collection, preclude from their generalizability. Moreover, there was a time interval of about 10 years between the collection of HRQOL data of French controls and those provided by both RCTs. This might also implicate some differences we were not able to take into account.

## Conclusion

Health-related quality of life of unshockable cardiac arrest survivors evaluated at 3 months was similar to ICU survivors and significantly lower than in individuals from general population, especially in the physical dimensions.

### Supplementary Information

**Additional file 1**: **Table S1**. Baseline characteristics of HYPERION participants according to their status atday-90. Data are shown as n(%) and median [interquartile] and compared using Pearson'schi-square and Kruskal-Wallis test for categorical and continuous variables, respectively.. **Table S2**. Baseline characteristics of AWARE participants according to their status at day-90.Data are shown as n(%) and median [interquartile] and compared using Pearson's chi-squareand Kruskal-Wallis test for categorical and continuous variables, respectively. **Table S3**. Baseline characteristics of respondents of the national French questionnaire accord-ing to the availability of HRQOL data. Data are shown median [interquartile] and comparedusing Mann-Whitney test. **Table S4**. Comparison of HRQOL data according to the randomization arm of the HYPER-ION trial. **Table S5**. Comparaison of Utstein variables according to randomization arm of the HYPER-ION trial. **Figure S1**. Proportion of missing values for each dimension of the SF-36 questionnaire in thethree sub-groups of patients included in the analysis. Each dimension is shown as a row.

## Data Availability

Data were available on reasonable request to Dr Jean-Baptiste Lascarrou.

## Appendix

We thank the HYPERION and AWARE study group investigators:

*HYPERION study*

MERDJI Hamid, Médecine Intensive Réanimation, CHRU, STRASBOURG, France ; GRILLET Guillaume, Médecine Intensive Réanimation, LORIENT, FRANCE ; GIRARDIE Patrick, Médecine Intensive Réanimation, LILLE, France ; COUPEZ Elisabeth, Médecine Intensive Réanimation, CLERMOND FERRAND, France ; DEQUIN Pierre-François, Médecine Intensive Réanimation, TOURS, France ; BOULAIN Thierry, Médecine Intensive Réanimation, ORLEANS, France ; FRAT Jean-Pierre, Médecine Intensive Réanimation, POITIERS, France ; ASFAR Pierre, Médecine Intensive Réanimation, ANGERS, France ; PICHON Nicolas, Médecine Intensive Réanimation, LIMOGES, France ; LANDAIS Mickael, Médecine Intensive Réanimation, LE MANS, France ; PLANTEFEVE Gaëtan, Médecine Intensive Réanimation, ARGENTEUIL, France ; QUENOT Jean-Pierre, Médecine Intensive Réanimation, DIJJON, France ; CHAKARIAN Jean-Charles, Médecine Intensive Réanimation, ROANNE, France ; SIRODOT Michel, Médecine Intensive Réanimation, ANNECY, France ; LEGRIEL Stéphane, Médecine Intensive Réanimation, VERSAILLES, France ; LETHEULLE Julien, Médecine Intensive Réanimation, SAINT BRIEUX, France ; THEVENIN Didier, Médecine Intensive Réanimation, LENS, France ; DESACHY Arnaud, Médecine Intensive Réanimation, ANGOULEME, France ; DELAHAYE Arnaud, Médecine Intensive Réanimation, RODEZ, France ; BOTOC Vlad, Médecine Intensive Réanimation, SAINT MALO, France ; VIMEUX Sylvie, Médecine Intensive Réanimation, MONTAUBAN, France ; MARTINO Frederic, Médecine Intensive Réanimation, POINT A PITRE, France

*AWARE study*

ABOAB Jérôme, Hôpital Raymond Poincaré, Réanimation, GARCHES, France ; ALIANE Jugurtha, CHU Gabriel Montpied, Réanimation Médicale Polyvalente, CLERMONT-FERRAND, FRANCE ; AISSAOUI Nadia, HEGP, PARIS, FRANCE ; ANNANE Djillali, Hôpital Raymond Poincaré, Réanimation, GARCHES, France ; AU Siu-Ming, Hôpital Ambroise Paré, Réanimation Médicale, BOULOGNE BILLANCOURT, FRANCE ; AUDOIN Corinne; Clinique des Cèdres, BLAGNAC, France ; AZOULAY Elie, Hôpital Saint Louis, Réanimation Médicale, PARIS, FRANCE ; BAUDEL Jean-Luc ; Médecine Intensive Réanimation, CHU Saint Antoine PARIS, France ; BODET-CONTENTIN Laetitia, CHRU de Tours ;Médecine Intensive Réanimation, TOURS, France ; BOULAIN Thierry, CHR d’Orléans Hôpital de la Source, Réanimation Médicale Polyvalente, ORLEANS, France ; BROUARD Florence; CHR René Dubost, PONTOISE, France ; CAMBONIE Alexandre; Médecine Intensive Réanimation, POITIERS, France ;CAMILATTO Isabelle; Médecine Intensive Réanimation, STRASBOURG, France ;CARTEAUX Guillaume, CHU Henri Mondor, Réanimation Médicale, CRETEIL, FRANCE ; CHERGUI Karim, CH Sud Francilien, Site Corbeil, Réanimation Polyvalente, CORBEIL-ESSONES, France ;CHOUQUER Renaud, CH de la Région d’Annecy, Réanimation Polyvalente, PRINGY, France ; CLAVIER Hervé, Institut Mutualiste Montsouris, Réanimation Polyvalente, PARIS, France ;CLEOPHAX Cédric, Hôpital René Dubos, Réanimation Médico-Chirurgicale, PONTOISE, France ; CRAVOISY-POPOVIC Aurélie, Hôpital Central, Réanimation Médicale, NANCY, France ; DAS Vincent; Médecine Intensive Réanimation, MONTREUIL, France ; DA SILVA Daniel, Centre Hospitalier de St Denis Hôpital de la Fontaine, Réanimation Médico-Chirurgicale, SAINT-DENIS, France ; DAURIAC Sandie, Hôpital Jean Bernard; CH de Valenciennes, Réanimation Polyvalente, VALENCIENNES, France ; DE JONGHE Bernard, CH de Poissy, Réanimation médico-chirurgicale, POISSY, France ; DESMARETZ Jean-Luc, CH Armentières, Réanimation, ARMENTIERES, France ; DEVOS Nicolas; Réanimation, Clinique de l’Europe, ROUEN, France ; DEYE Nicolas; Médecine Intensive Réanimation, Hopital Lariboisière, PARIS, France ; DONETTI Laurence, GHIRM MONTFERMEIL, Réanimation, MONTFERMEIL, France ; DUGUET Alexandre, CHU La Pitié Salpêtrière, Pneumologie et Service de Réanimation, PARIS, France ; ENA Sébastien, CH de Rodez; Hôpital Jacques Puel, Réanimation, RODEZ, France ; EHRMANN Stephan (writing committee); CHRU Bretonneau, TOURS, FRANCE ; FOUREL Didier, HIA Clermont Tonnerre, Fédération Anesthésie Réanimation Urgences, Réanimation polyvalente, BREST, FRANCE ; GANSTER Frédérique; Médecine Intensive Réanimation, MULHOUSE, France ; GIRARDIE Patrick, Hôpital Roger Salengro, Réanimation Polyvalent, LILLE, France ; GIRAULT Christophe, CHU de Rouen; Hôpital Charles Nicolle, Réanimation Médicale, ROUEN, France ; GOURDIN Emmanuelle ; Médecine Intensive Réanimation, MONTREUIL, France ; GRIMALDI David; Intensive Care Unit, ERASME, BRUXELLES, Belgique ; GROS Antoine; Médecine Intensive Réanimation, VERSAILLES, France ; GUERIN Laurent, CHU Kremlin-Bicêtre, Réanimation Médicale, LE KREMLIN-BICETRE, France ; HAMZAOUI Olfa; Médecine Intensive Réanimation, CLAMART, France ; HERNU Romain, Groupe Hospitalier Edouard Herriot, Réanimation Médicale, LYON, France ; HICTER Jean-François, Clinique Belledonne, Réanimation polyvalente, SAINT MARTIN D’HERES, France ; HYVERNAT Hervé, Hôpital l’Archet, Réanimation Médicale, NICE, France ;  JACOBS Frédéric; Médecine Intensive Réanimation, CLAMART, France ; JACQUES Thierry, Polyclinique de Gentilly, Réanimation, NANCY, France ; KIMMOUN Antoine; Médecine Intensive Réanimation, NANCY, France ;LACHERADE Jean-Claude; Médecine Intensive Réanimation, LA ROCHE SUR YON, France ; LAMBERMONT Bernard; Intensive Care Unit, LIEGES, BELGIUM ; LATERRE Pierre-François; Medical-surgical intensive care unit, Saint Luc University Hospital, Université Catholique de Louvain, Brussels, BELGIUM ; LEGRIEL Stéphane ; Médecine Intensive Réanimation, VERSAILLES, France ; LIAUDET Lucas ; Service de Réanimation, LAUSANNE, SWITZERLAND ; LU Qin, CHU La Pitié Salpêtrière, Réanimation polyvalente, PARIS, France ; LUYT Charles-Edouard; Médecine Intensive Réanimation, La Pitié Salpétrière, PARIS, France ; MARJOT-ZIMBACCA France, CH de St Brieuc, Réanimation Polyvalente, SAINT-BRIEUC, France ; MERAT Stéphane, HIA Bégin, Réanimation, SAINT-MANDE, France ; MERCAT Alain, CHU d’Angers, Réanimation Médicale, ANGERS, France ; MICHEL Philippe ; CHR René Dubost, PONTOISE, France ; MIRA Jean-Paul ; Médecine Intensive Réanimation Hopital Cochin, PARIS, France ; MOHEBBI AMOLI Abolfazl, Hôpital Privé d’Antony, Réanimation Polyvalente, ANTONY, France ; MONNET Xavier; Médecine Intensive Réanimation, KREMLIN-BICETRE, France ; MULLER Grégoire; CHR, ORLEANS, France ; OBBEE Philippe, Centre Hospitalier de Mâcon, Réanimation Polyvalente, MACON, France ; PIAGNERELLI Michael; Department of Intensive Care Experimental Medicine Laboratory, Centre Hospitalier Universitaire, Charleroi, Belgium ; PICARD Walter, CH François Mitterand, Réanimation Polyvalente, PAU, France ; PLANTEFEVE Gaëtan, CH Victor Dupouy, Réanimation Polyvalente, ARGENTEUIL, France ; PLOUVIER Fabienne, Hôpital Saint-Esprit, Réanimation Polyvalente, AGEN, France ; PRAT Gwénaël, CHRU Cavale Blanche, Réanimation Médicale, BREST, France ; QUENTIN Charlotte, CH de Saint-Malo, Réanimation Polyvalente, SAINT-MALO, France ; REIGNIER Jean, CHD Les Oudairies, Réanimation Polyvalente, LA ROCHE SUR YON, France ; REUTER Danielle, Hôpital Saint Louis, Réanimation Médicale, PARIS, France ; RICARD Jean-Damien ; Médecine Intensive Réanimation Louis Mourier, COLOMBES, France ; RIGAUD Jean-Philippe, CH Diepp, Réanimation Polyvalente, DIEPPE, France ; ROBERT René, CHU de Poitier, Réanimation Médicale, POITIERS, France ; SCHENCK Maleka, Hôpital de Hautepierre, Réanimation Médicale, STRASBOURG, France ; STOCLIN Annabelle, Institut Gustave Roussy, Réanimation Polyvalente - USCM, VILLEJUIF, France ; SOUMER Alexis, Hôpital Foch, Réanimation polyvalente, SURESNES, France ; TROCHE Gilles, CH de Versailles - Site André Mignot, Réanimation Médico-chirurgicale, LE CHESNAY, France ; VINCENT François, CHIC, MONTFERMEIL, France ; VIVIER Emmanuel, Centre Hospitalier St Joseph St Luc, Réanimation Polyvalente, LYON, France ;
