# Supplementary material for: Health-related quality of life in critically ill survivors: specific impact of cardiac arrest in non-shockable rhythm
Source: Ann Intensive Care. 2021 Oct 24;11:150. doi: 10.1186/s13613-021-00939-w (PMC8542521; doi:10.1186/s13613-021-00939-w)
Supplement: Supplementary file 1 — Additional file 1: Table S1. Baseline characteristics of HYPERION participants according to their status atday-90. Data are shown as n(%) and median [interquartile] and compared using Pearson'schi-square and Kruskal-Wallis test for categorical and continuous variables, respectively.. Table S2. Baseline characteristics of AWARE participants according to their status at day-90.Data are shown as n(%) and median [interquartile] and compared using Pearson's chi-squareand Kruskal-Wallis test for categorical and continuous variables, respectively. Table S3. Baseline characteristics of respondents of the national French questionnaire accord-ing to the availability of HRQOL data. Data are shown median [interquartile] and comparedusing Mann-Whitney test. Table S4. Comparison of HRQOL data according to the randomization arm of the HYPER-ION trial. Table S5. Comparaison of Utstein variables according to randomization arm of the HYPER-ION trial. Figure S1. Proportion of missing values for each dimension of the SF-36 questionnaire in thethree sub-groups of patients included in the analysis. Each dimension is shown as a row. [file 13613_2021_939_MOESM1_ESM.pdf]

Health-related quality of life in critically ill survivors: specific impact of cardiac arrest in non-shockable rhythm
Supplementary material

September 14, 2021

	Available HRQOL (n=72)	Expired within d90 (n=478)	Lost of follow-up (n=31)	P value
Age, y	64 [56,72]	67 [57,76]	67 [55,76]	0.26
Male gender	49 (68.1)	307 (64.2)	17 (54.8)	1.00
SAPS2 score	73 [55,80]	74 [65,84]	76 [60,87.5]	0.11
Charlson score	1 [0,3]	1 [0,3]	3 [0,4]	0.38
Utstein criteria				
Presence of witness	69 (95.8)	447 (93.5)	31 (100)	0.27
Bystander CPR	58 (80.6)	324 (67.8)	25 (80.6)	0.04
Collapse to BLS	0 [0,8.4]	5.6 [0,14]	0 [0,14]	<0.001
Collapse to ROSC	8 [4,12]	16 [8,22.4]	8 [5.2,12]	<0.001
CAHP score	170 [143,187]	197 [173,218]	170 [150,190]	
Randomization group				0.69
TTM 33	38 (52.8)	247 (51.7)	12 (38.7)	
TTM 37	34 (47.2)	231 (48.3)	19 (61.3)	

Table 1: Baseline characteristics of HYPERION participants according to their status at day-90. Data are shown as n(%) and median [interquartile] and compared using Pearson's chi-square and Kruskal-Wallis test for categorical and continuous variables, respectively.

	Available HRQOL (n=307)	Unavailable HRQOL (n=355)	Expired within day-90 (n=475)	Lost of follow-up (n=24)	P value
Age, y	62 [50,71]	68 [60,78]	72 [63,79]	68 [62,75]	<0.001
Male gender	184 (59.9)	221 (62.3)	339 (71.4)	20 (83.3)	0.001
SAPS2 score	45 [35,56]	50 [40,61]	58 [47,73]	57 [45,64]	<0.001
SOFA score	8 [6,10]	9 [7,11]	11 [8,13]	8.5 [6,11]	<0.001
ARDS	99 (32.2)	97 (27.3)	169 (35.7)	2 (8.3)	0.005
Septic shock	142 (46.3)	187 (52.7)	310 (65.3)	13 (54.2)	<0.001
Randomization group					0.35
Controls	141 (45.9)	178 (50.1)	249 (52.4)	13 (54.2)	
Oversed. prevention	166 (54.1)	177 (49.9)	226 (47.6)	11 (45.8)	

Table 2: Baseline characteristics of AWARE participants according to their status at day-90. Data are shown as n(%) and median [interquartile] and compared using Pearson's chi-square and Kruskal-Wallis test for categorical and continuous variables, respectively.

	Available HRQOL data n=20,574	Unavailable HRQOL data n=5,537	P value
Age, y	44 [32,56]	55 [40,70]	<0.001
Male gender	10,899 (53.0)	2,884 (53.8)	0.267

Table 3: Baseline characteristics of respondents of the national French questionnaire according to the availability of HRQOL data. Data are shown median [interquartile] and compared using Mann-Whitney test

	TTM 33	TTM37	p-value
Phys. funct.	75.0 [60.0;88.8]	35.0 [11.2;78.4]	0.017
Role phys.	25.0 [0.00;75.0]	0.00 [0.00;43.8]	0.111
Bos. pain	79.0 [41.0;100]	66.5 [41.0;100]	0.360
Gen health	62.0 [52.0;80.8]	51.0 [35.0;62.7]	0.019
Vitality	52.5 [36.2;65.0]	40.0 [20.0;55.0]	0.202
Soc. funct.	56.2 [40.6;75.0]	43.8 [25.0;59.4]	0.019
Role emot.	66.7 [0.00;100]	33.3 [0.00;100]	0.665
Ment. health	68.0 [60.0;79.0]	64.0 [44.0;76.0]	0.217
PCS	44.3 [34.4;50.4]	36.6 [27.1;41.7]	0.011
MCS	44.1 [36.3;53.6]	44.8 [31.2;52.1]	0.565

Table 4: Comparison of HRQOL data according to the randomization arm of the HYPERION trial

	TTM33	TTM37	p-value
SAPS2 score	73.0 [63.5;84.0]	75.0 [63.0;85.0]	0.636
Age,y	67.0 [56.2;76.0]	67.0 [57.0;76.0]	0.855
Male gender	185 (65.1%)	188 (63.3%)	0.707
Witnessed CA	274 (96.5%)	273 (92.2%)	0.042
Bystander CPR	200 (73.3%)	207 (75.5%)	0.607
Location of CA			0.089
home	138 (48.6%)	157 (52.9%)	
hospital	73 (25.7%)	86 (29.0%)	
public setting	73 (25.7%)	54 (18.2%)	
Collapse to CPR, min	2.00 [0.00;5.00]	1.00 [0.00;5.00]	0.255
CPR to ROSC, min	15.0 [10.0;25.0]	18.0 [10.0;26.0]	0.471

Table 5: Comparaison of Utstein variables according to randomization arm of the HYPERION trial

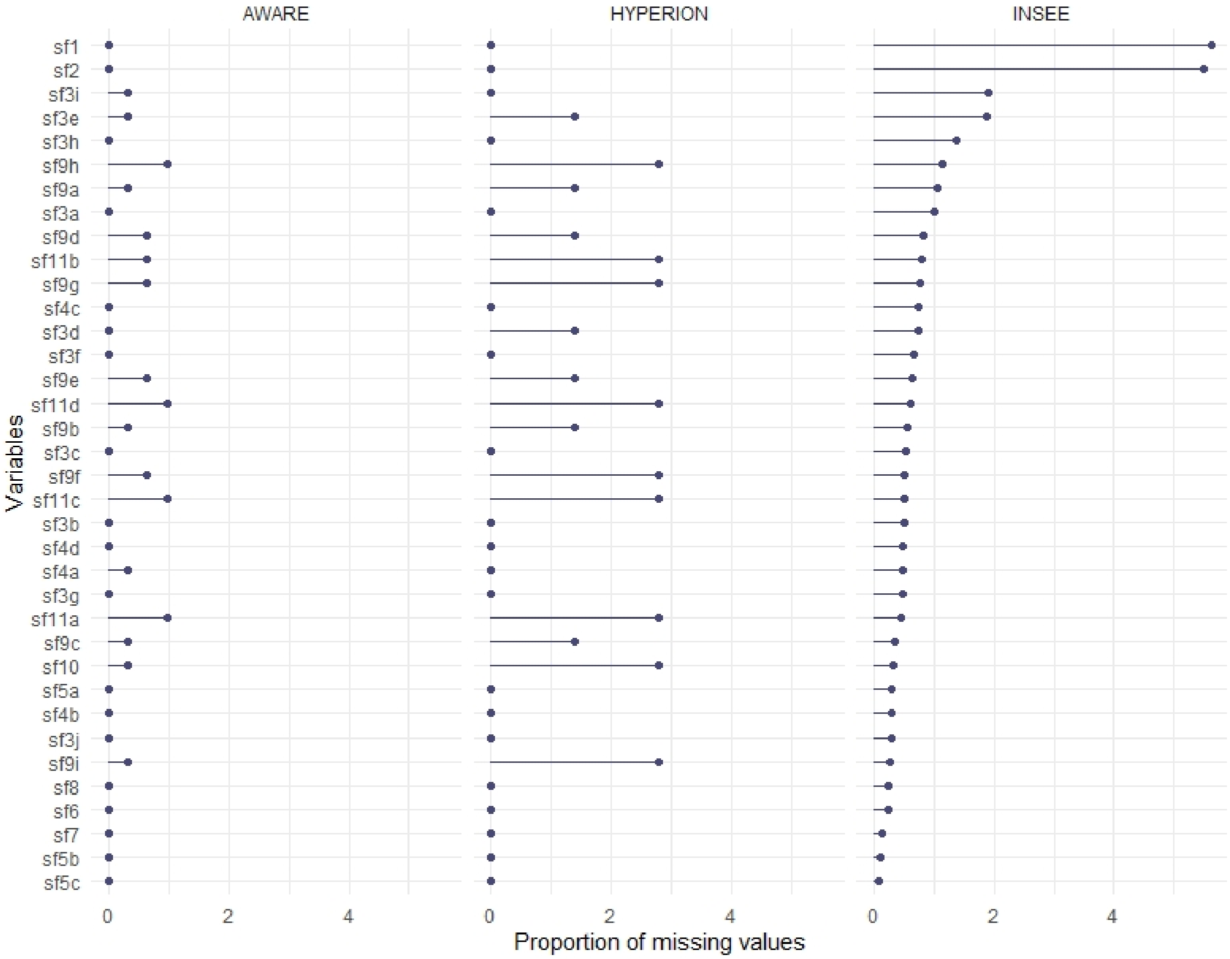

Figure 1: roportion of missing values for each dimension of the SF-36 questionnaire in the three sub-groups of patients included in the analysis. Each dimension is shown as a row.
